# Association of Serum Ferritin and Cytokine Profile With Severity of Acute Cholecystitis in Patients Undergoing Laparoscopic Cholecystectomy

**DOI:** 10.7759/cureus.116311

**Published:** 2026-09-15

**Authors:** Shafqat Noor, Rafique Ahmed, Sumreen, Tahir Aslam, Sarah Azhar, Alina Mehwish

**Affiliations:** 1 General Surgery, Hunter New England Health, John Hunter Hospital, Newcastle, AUS; 2 General Surgery, Rustaq Hospital, Al-Rustaq, OMN; 3 General Surgery, Shaheed Mohterma Benazir Bhuto Medical College Lyari, Karachi, PAK; 4 Thoracic Surgery, Fatima Jinnah Institute of Chest Diseases, Quetta, PAK; 5 Pathology, Baqai Medical College, Karachi, PAK; 6 Microbiology, Jinnah Medical College, Peshawar, PAK

**Keywords:** acute cholecystitis, disease severity, interleukin-6, serum ferritin, tumor necrosis factor-alpha

## Abstract

Background: Acute cholecystitis is a common surgical emergency. Early assessment of disease severity is important for safe laparoscopic cholecystectomy in routine hospital practice in Pakistan. Serum ferritin and inflammatory cytokines may reflect the intensity of systemic inflammation. This study assessed their association with acute cholecystitis severity.

Methods: This observational study was conducted at Shaheed Mohtarma Benazir Bhutto Medical College, Lyari, Pakistan, over six months, starting in March 2025. Ninety-four adults with acute cholecystitis undergoing laparoscopic cholecystectomy were included by consecutive sampling. Patients were classified into Grade I, II, and III according to the Tokyo Guidelines 2018. Serum ferritin, interleukin (IL)-6, tumor necrosis factor-alpha (TNF-α), and IL-10 were measured before surgery. Complete blood count, C-reactive protein (CRP), bilirubin, alanine aminotransferase (ALT), and aspartate aminotransferase (AST) were also recorded. Correlation and multivariable regression analyses were performed.

Results: Of 94 patients, 38 (40.4%) had Grade I, 39 (41.5%) Grade II, and 17 (18.1%) Grade III disease. Median ferritin increased from 146 ng/mL in Grade I to 428 ng/mL in Grade III (p<0.001). IL-6 increased from 13.2 to 56.8 pg/mL, while TNF-α increased from 10.8 to 39.5 pg/mL (both p<0.001). IL-10 increased from 6.4 to 14.2 pg/mL (p=0.002). Ferritin correlated with disease grade (r=0.64, p<0.001), while IL-6 showed the strongest correlation (r=0.72, p<0.001). After adjustment, ferritin, IL-6, and TNF-α remained independently associated with severe disease.

Conclusion: Serum ferritin and inflammatory cytokines, particularly IL-6 and TNF-α, were associated with increasing acute cholecystitis severity. Their use alongside routine inflammatory markers may help identify high-risk patients and support closer monitoring and better surgical planning before early laparoscopic cholecystectomy in routine hospital practice in Pakistan.

## Introduction

Acute cholecystitis is a common condition seen in surgical emergency departments. It usually develops due to obstruction of the cystic duct by a gallstone [1]. The obstruction causes gallbladder distension, inflammation, and sometimes infection. In severe cases, the disease can progress to gangrene, perforation, sepsis, and organ dysfunction. Early recognition of severe disease is therefore important for proper treatment and safe surgery [2].

The Tokyo Guidelines 2018 provide a standard method for diagnosis and severity assessment of acute cholecystitis. The disease is divided into Grade I, Grade II, and Grade III. Grade I represents mild disease [3]. Grade II indicates more marked local inflammation. Grade III is associated with organ dysfunction. This grading system has been validated and is associated with important outcomes such as hospital stay and conversion from laparoscopic to open surgery [4,5].

Routine laboratory markers such as white blood cell count and C-reactive protein (CRP) are useful indicators of inflammation; however, these markers may not fully explain the systemic inflammatory response [6]. Cytokines are involved in the development and control of inflammation. Interleukin (IL)-6 is an important inflammatory cytokine. It stimulates the acute-phase response and may increase with increasing disease severity [7]. Recent research has reported higher IL-6 levels in patients with moderate and severe acute cholecystitis [8].

Tumor necrosis factor-alpha (TNF-α) is another important pro-inflammatory cytokine. It helps activate immune cells and increases the inflammatory response [9]. Studies in acute calculous cholecystitis have reported increased TNF-α levels. Higher TNF-α has also been linked with longer hospital stay [10]. IL-10 has an anti-inflammatory role. Its increase may represent the body's response to control excessive inflammation. Previous research has shown that IL-10 and IL-6 can help assess the severity and progression of acute calculous cholecystitis [11]. Serum ferritin is mainly known as a marker of iron storage. It is also an acute-phase reactant and can increase during inflammation. Therefore, its level may reflect the intensity of inflammatory activity [12].

The relationship between ferritin, cytokines, and acute cholecystitis severity is less studied than routine inflammatory markers. Studying ferritin together with IL-6, TNF-α, and IL-10 may provide a better understanding of the inflammatory response in acute cholecystitis. The present study was conducted to assess the association of serum ferritin and cytokine profile with the severity of acute cholecystitis in patients undergoing laparoscopic cholecystectomy. The study also examined their relationship with routine inflammatory markers and selected clinical and operative outcomes.

## Materials and methods

This was an observational study conducted at the Shaheed Mohtarma Benazir Bhutto Medical College Lyari, Karachi, Pakistan, over a period of six months, starting in March 2025. The study was reported according to the Strengthening the Reporting of Observational Studies in Epidemiology (STROBE) guidelines [13]. The study was approved by the Institutional Review Board of Shaheed Mohtarma Benazir Bhutto Medical College (reference number IRB/2025/076/SMBBMC, dated February 18, 2025). Written informed consent was obtained from all participants before enrollment.

Study population

Eligibility Criteria

Patients diagnosed with acute cholecystitis and planned for laparoscopic cholecystectomy were included. The diagnosis was based on clinical findings, laboratory investigations, and abdominal ultrasonography. Patients aged 18 years or above were eligible. Patients with chronic liver disease, chronic kidney disease, active malignancy, autoimmune disease, acute or chronic pancreatitis, severe infection other than acute cholecystitis, and those receiving corticosteroids or immunosuppressive drugs were excluded. Pregnant women and patients who refused consent were also excluded. Consecutive sampling was used, and all eligible patients presenting during the study period were recruited until the required sample size was reached.

Sample Size Calculation

The sample size was calculated using the formula for correlation studies, considering an expected correlation coefficient of 0.30 between serum ferritin and disease severity, a 5% level of significance, and 80% study power [14]. The calculated minimum sample size was 85 patients. To allow for approximately 10% incomplete data or withdrawals, 94 patients were recruited.

Data collection

Demographic data, including age and sex, were recorded. Clinical findings included fever, right upper abdominal pain, nausea, vomiting, pulse rate, blood pressure, and duration of symptoms. Routine laboratory tests included complete blood count, total leukocyte count, neutrophil count, CRP, liver function tests, and serum bilirubin. Blood samples were collected before surgery and before administration of perioperative blood products or other major interventions.

Measurements/assessments

For serum ferritin measurement, 3 mL of venous blood was collected in a plain tube. The sample was allowed to clot and was centrifuged at approximately 3000 rpm for 10 minutes. The serum was separated and stored at −20°C until analysis. Serum ferritin was measured using a commercially available enzyme-linked immunosorbent assay (ELISA) kit according to the manufacturer's instructions (Elabscience, Wuhan, Hubei, China). Cytokines including IL-6, TNF-α, and IL-10 were measured from the same serum sample using commercially available ELISA kits. Absorbance was measured with a standard microplate reader, and concentrations were calculated from the standard curves provided with each kit. Samples were analyzed in duplicate to improve measurement reliability.

The severity of acute cholecystitis was assessed according to the Tokyo Guidelines 2018 [3]. Patients were classified into Grade I, Grade II, or Grade III according to the specified clinical, laboratory, and organ dysfunction criteria [15]. Operative findings, duration of surgery, conversion to open surgery, postoperative complications, and length of hospital stay were also recorded. These variables were used as secondary indicators of disease severity.

Potential confounding factors included age, sex, body mass index (BMI), diabetes mellitus, hypertension, smoking status, duration of symptoms before admission, and baseline white blood cell count and CRP level. These factors were recorded and considered during statistical analysis. Data were checked for completeness before analysis. Records with missing values for the primary outcome or main exposure were excluded from the relevant analysis. For other variables, the available data were analyzed, and the number of missing observations was reported.

Data analysis

Data were analyzed using IBM SPSS Statistics for Windows, version 25.0 (IBM Corp., Armonk, New York, United States). Continuous variables were tested for normality using the Shapiro-Wilk test. Normally distributed data were presented as mean ± standard deviation (SD), while non-normally distributed data were presented as median with interquartile range (IQR). Categorical variables were presented as frequencies and percentages. Differences in ferritin and cytokine levels among the three severity grades were assessed using one-way analysis of variance for normally distributed data and the Kruskal-Wallis test for non-normally distributed data. The chi-square test was used for categorical variables. Pearson or Spearman correlation analysis was used to assess the relationship between serum ferritin, cytokine levels, and severity indicators, according to data distribution. Multivariable regression analysis was performed to assess the independent association of ferritin and cytokine levels with severe acute cholecystitis after adjustment for relevant confounders. A two-sided p-value of <0.05 was considered statistically significant.

## Results

A total of 94 patients with acute cholecystitis who underwent laparoscopic cholecystectomy were included in the study. The patients were classified according to the Tokyo Guidelines 2018. There were 38 (40.4%) patients with Grade I disease, 39 (41.5%) with Grade II disease, and 17 (18.1%) with Grade III disease. The mean age of the participants was 45.8 ± 13.2 years. Female patients were more common than male patients. The mean duration of symptoms before admission was 3.1 ± 1.8 days. Baseline demographic and clinical characteristics are presented in Table 1.

**Table 1:** Demographic and clinical characteristics of the study participants Disease severity was classified according to the Tokyo Guidelines 2018 [3]. One-way analysis of variance (ANOVA) was used for continuous variables and Pearson's chi-square (χ²) test was used for categorical variables. BMI, body mass index; SD, standard deviation.

Variable	Overall (n=94)	Grade I (n=38)	Grade II (n=39)	Grade III (n=17)	Test statistic	p-value
Age (years), mean ± SD	45.8 ± 13.2	41.9 ± 11.8	46.7 ± 12.9	52.4 ± 14.1	F = 4.19	0.018
Male, n (%)	37 (39.4)	13 (34.2)	15 (38.5)	9 (52.9)	χ² = 1.75	0.421
Female, n (%)	57 (60.6)	25 (65.8)	24 (61.5)	8 (47.1)		
BMI (kg/m²), mean ± SD	27.1 ± 3.9	26.4 ± 3.5	27.2 ± 3.8	28.6 ± 4.4	F = 1.98	0.124
Diabetes mellitus, n (%)	21 (22.3)	5 (13.2)	10 (25.6)	6 (35.3)	χ² = 4.43	0.108
Hypertension, n (%)	24 (25.5)	6 (15.8)	11 (28.2)	7 (41.2)	χ² = 5.00	0.083
Smoking, n (%)	19 (20.2)	5 (13.2)	9 (23.1)	5 (29.4)	χ² = 2.74	0.254
Duration of symptoms (days), mean ± SD	3.1 ± 1.8	2.3 ± 1.1	3.3 ± 1.5	4.6 ± 2.5	F = 12.65	<0.001
Fever, n (%)	56 (59.6)	16 (42.1)	25 (64.1)	15 (88.2)	χ² = 11.62	0.003
Right upper abdominal pain, n (%)	89 (94.7)	35 (92.1)	37 (94.9)	17 (100)	χ² = 1.40	0.497
Nausea/vomiting, n (%)	62 (66.0)	21 (55.3)	28 (71.8)	13 (76.5)	χ² = 3.37	0.184
Pulse rate (beats/min), mean ± SD	91.6 ± 11.4	87.8 ± 8.9	92.4 ± 10.2	101.5 ± 13.6	F = 10.20	<0.001

Patients with higher disease grades had a greater duration of symptoms and higher pulse rates. Fever was also more frequent in Grade III disease. Age showed a significant difference among the three groups, whereas sex, BMI, diabetes, hypertension, and smoking status were not significantly different. Laboratory findings showed progressive increases in inflammatory markers with increasing disease severity. The mean total leukocyte count and neutrophil count were significantly higher in Grade III patients. Serum CRP also increased markedly across the severity groups. Mild elevations in serum bilirubin and liver enzymes were observed in patients with more severe disease. The laboratory findings are shown in Table 2.

**Table 2:** Laboratory findings according to severity of acute cholecystitis One-way analysis of variance (ANOVA) was used for normally distributed continuous variables, while the Kruskal–Wallis test was used for CRP. CRP, C-reactive protein; ALT, alanine aminotransferase; AST, aspartate aminotransferase; IQR, interquartile range; SD, standard deviation.

Parameter	Overall (n=94)	Grade I (n=38)	Grade II (n=39)	Grade III (n=17)	Test statistic	p-value
Total leukocyte count (×10⁹/L), mean±SD	13.2 ± 4.1	10.8 ± 2.6	13.7 ± 3.2	18.1 ± 4.3	F = 30.92	<0.001
Neutrophils (%), mean±SD	78.6 ± 8.4	73.8 ± 6.1	80.2 ± 6.8	87.4 ± 6.2	F = 27.70	<0.001
CRP (mg/L), median (IQR)	72 (38–126)	42 (25–67)	82 (51–126)	168 (112–231)	H = 28.64	<0.001
Total bilirubin (mg/dL), mean±SD	1.21 ± 0.48	1.04 ± 0.31	1.23 ± 0.42	1.55 ± 0.69	F = 7.84	0.006
ALT (U/L), mean±SD	43.7 ± 18.5	37.8 ± 12.6	44.5 ± 16.9	54.8 ± 27.1	F = 5.50	0.021
AST (U/L), mean±SD	41.9 ± 17.8	36.4 ± 12.1	42.7 ± 15.6	53.6 ± 25.8	F = 6.29	0.014
Hemoglobin (g/dL), mean±SD	12.8 ± 1.6	12.9 ± 1.5	12.7 ± 1.7	12.5 ± 1.8	F = 0.37	0.682

Serum ferritin and cytokine concentrations increased with increasing severity of acute cholecystitis. The median ferritin level was 146 ng/mL in Grade I disease and increased to 428 ng/mL in Grade III disease. Similarly, IL-6 and TNF-α showed marked increases in Grade III patients. IL-10 also increased with disease severity, although the difference was less pronounced than that observed for IL-6 and TNF-α. These findings are presented in Table 3.

**Table 3:** Serum ferritin and cytokine levels according to severity of acute cholecystitis Differences among Grade I, Grade II, and Grade III groups were assessed using the Kruskal–Wallis test. IL, interleukin; TNF-α, tumor necrosis factor-alpha; IQR, interquartile range

Parameter	Overall (n=94), median (IQR)	Grade I (n=38), median (IQR)	Grade II (n=39), median (IQR)	Grade III (n=17)	Test statistic	p-value
Ferritin (ng/mL)	214 (126–326)	146 (92–208)	226 (151–321)	428 (302–586)	H = 24.86	<0.001
IL-6 (pg/mL)	24.8 (14.6–39.7)	13.2 (9.1–19.8)	27.6 (19.3–38.5)	56.8 (39.4–81.6)	H = 31.42	<0.001
TNF-α (pg/mL)	18.6 (11.4–28.7)	10.8 (7.2–16.1)	19.7 (13.8–27.4)	39.5 (27.1–52.8)	H = 27.63	<0.001
IL-10 (pg/mL)	8.7 (5.6–13.9)	6.4 (4.3–9.2)	9.1 (6.2–13.7)	14.2 (9.1–21.5)	H = 12.47	0.002

Postoperative and operative findings also differed according to disease severity. The mean operative time increased from 61.4 minutes in Grade I disease to 89.6 minutes in Grade III disease. Conversion to open surgery and postoperative complications were more frequent among patients with severe disease. Length of hospital stay was significantly longer in Grade III patients. The operative findings are shown in Table 4.

**Table 4:** Operative and postoperative outcomes according to severity of acute cholecystitis One-way analysis of variance (ANOVA) was used for operative time, with F = 17.97. Pearson's chi-square test or Fisher's exact test was used for categorical variables according to the expected cell frequencies. The Kruskal–Wallis test was used for length of hospital stay. IQR, interquartile range

Variable	Overall (n=94)	Grade I (n=38)	Grade II (n=39)	Grade III (n=17)	Test statistic	p-value
Operative time (minutes), mean ± SD	69.2 ± 18.7	61.4 ± 12.8	71.3 ± 16.5	89.6 ± 21.4	F = 17.97	<0.001
Conversion to open surgery, n (%)	7 (7.4)	1 (2.6)	3 (7.7)	3 (17.6)	χ² = 4.72	0.094
Postoperative complications, n (%)	13 (13.8)	2 (5.3)	5 (12.8)	6 (35.3)	χ² = 10.02	0.007
Length of hospital stay (days), median (IQR)	3 (2–5)	2 (2–3)	3 (2–4)	6 (4–8)	H = 22.81	<0.001

Correlation analysis showed a significant positive relationship between serum ferritin and the inflammatory cytokines. Ferritin had a strong positive correlation with IL-6 and moderate positive correlations with TNF-α and IL-10. Ferritin was also positively correlated with CRP, total leukocyte count, duration of symptoms, operative time, and hospital stay. IL-6 showed the strongest correlation with disease severity. The correlation results are presented in Table 5.

**Table 5:** Correlation of serum ferritin and cytokines with inflammatory and clinical severity indicators Pearson or Spearman correlation coefficients were used according to the distribution of the variables. CRP, C-reactive protein; IL-6, interleukin-6; TNF-α, tumor necrosis factor-alpha; IL-10, interleukin-10.

Variable	Ferritin r	p-value	IL-6 r	p-value	TNF-α r	p-value	IL-10 r	p-value
CRP	0.62	<0.001	0.71	<0.001	0.58	<0.001	0.36	<0.001
Total leukocyte count	0.51	<0.001	0.59	<0.001	0.48	<0.001	0.29	0.004
Duration of symptoms	0.43	<0.001	0.46	<0.001	0.39	<0.001	0.24	0.020
Operative time	0.48	<0.001	0.52	<0.001	0.44	<0.001	0.27	0.009
Hospital stay	0.51	<0.001	0.56	<0.001	0.46	<0.001	0.30	0.003
Disease severity grade	0.64	<0.001	0.72	<0.001	0.61	<0.001	0.35	<0.001

Pearson correlation was used for normally distributed variables, while Spearman correlation was used for non-normally distributed variables. All four biomarkers showed a significant positive association with disease severity. The strongest association was observed for IL-6, followed by ferritin and TNF-α.

For multivariable analysis, Grade III disease was considered severe acute cholecystitis and was compared with Grade I and Grade II disease. Age, sex, BMI, diabetes, hypertension, smoking, duration of symptoms, CRP, ferritin, IL-6, TNF-α, and IL-10 were considered as potential explanatory variables. After adjustment, serum ferritin, IL-6, and TNF-α remained significantly associated with severe acute cholecystitis. IL-6 showed the strongest independent association. The adjusted results are shown in Table 6.

**Table 6:** Multivariable logistic regression analysis of factors associated with severe acute cholecystitis Severe acute cholecystitis was defined as Grade III disease according to the Tokyo Guidelines 2018 [3]. Results are presented as adjusted odds ratios with 95% confidence intervals and p-values. The Wald χ² test was used to assess the independent association of each variable with severe acute cholecystitis. The corresponding Wald χ² statistics were obtained from the multivariable logistic regression model. OR, odds ratio; CI, confidence interval; χ², Wald chi-square test statistic; CRP, C-reactive protein; TNF-α, tumor necrosis factor-alpha; IL, interleukin.

Variable	Adjusted OR	95% CI	Wald χ²	p-value
Age (per 10-year increase)	1.28	1.01–1.63	4.18	0.041
Male sex	1.42	0.48–4.19	0.40	0.526
BMI (per 1 kg/m² increase)	1.07	0.96–1.20	1.50	0.221
Diabetes mellitus	1.63	0.55–4.82	0.79	0.376
Hypertension	1.51	0.52–4.42	0.58	0.448
Smoking	1.31	0.43–3.96	0.23	0.630
Duration of symptoms (per day)	1.34	1.05–1.71	5.48	0.019
CRP (per 10 mg/L increase)	1.08	1.02–1.15	6.48	0.011
Ferritin (per 100 ng/mL increase)	1.47	1.16–1.87	9.42	0.002
IL-6 (per 10 pg/mL increase)	1.82	1.32–2.51	14.86	<0.001
TNF-α (per 10 pg/mL increase)	1.51	1.12–2.04	7.30	0.007
IL-10 (per 5 pg/mL increase)	1.19	0.91–1.56	1.63	0.202

## Discussion

The findings of the present study suggest that serum ferritin and inflammatory cytokines may help to identify patients with more severe acute cholecystitis. This is clinically important because the severity of acute cholecystitis can affect the difficulty of laparoscopic surgery, postoperative complications, and hospital stay. The Tokyo Guidelines classify acute cholecystitis into Grade I, II, and III according to the extent of inflammation and organ dysfunction. This grading has also been linked with hospital stay and conversion from laparoscopic to open surgery [15].

In our study, older age, longer duration of symptoms, fever, and higher pulse rate were more common in patients with severe disease. These findings are reasonable because prolonged inflammation can cause a greater local and systemic inflammatory response. Duration of more than 72 hours and high white blood cell count are also recognized features of more advanced acute cholecystitis in the Tokyo Guidelines [3]. The higher total leukocyte count and neutrophil percentage in Grade III patients further support this inflammatory response. Recent research has also shown that neutrophil count is higher in moderate-to-severe acute cholecystitis and can help in assessing disease progression [16].

CRP showed a clear rise with increasing disease severity in our study. Grade III patients had much higher CRP than Grade I patients. It is an acute-phase marker and is commonly increased during gallbladder inflammation. A recent cohort study also reported that CRP was strongly associated with complicated acute cholecystitis and had good ability to identify local complications [17]. Therefore, the higher CRP in our severe group is expected and supports the validity of our severity classification. Mild increases in bilirubin, alanine aminotransferase (ALT), and aspartate aminotransferase (AST) were also seen in severe disease. This may be related to greater gallbladder inflammation and temporary involvement of the biliary system.

The most important finding of our study was the progressive increase in serum ferritin from Grade I to Grade III disease. Ferritin increased from a median of 146 ng/mL in Grade I to 428 ng/mL in Grade III. Ferritin is usually considered a marker of body iron stores, but it also acts as an acute-phase reactant. Its level can rise during infection and inflammation even when total body iron is not increased. Inflammation can increase ferritin production and reduce the release of iron from tissues [12]. This response is considered part of the body's defence against infection. Thus, the high ferritin level in our Grade III patients may reflect the stronger systemic inflammatory response rather than an increase in iron stores alone.

The positive correlation between ferritin and CRP, total leukocyte count, duration of symptoms, and Tokyo severity grade further supports this explanation. Ferritin also showed a positive correlation with IL-6 and TNF-α. This relationship has biological support because ferritin is closely linked with inflammatory cytokines, particularly IL-6 and TNF-α. Previous research has reported positive associations between ferritin, IL-6, TNF-α, and CRP [18]. Therefore, ferritin may provide an additional simple marker of the inflammatory burden in acute cholecystitis.

Among the cytokines, IL-6 showed the strongest association with disease severity. Its level increased markedly from Grade I to Grade III and had the highest correlation with the severity grade. IL-6 is an important mediator of the acute inflammatory response. It stimulates several acute-phase reactions and can increase during severe tissue inflammation. Recent studies in acute cholecystitis have reported significantly higher IL-6 levels in moderate-to-severe disease. A 2024 study also found that IL-6 was useful for assessing acute cholecystitis severity, and its combination with another inflammatory marker improved the predictive value [16]. These findings are in agreement with our results.

TNF-α also increased significantly with disease severity. It is an important pro-inflammatory cytokine and is involved in activation of immune cells and amplification of local inflammation. Earlier work in patients with acute calculous cholecystitis reported increased circulating TNF-α together with IL-6 and IL-10 before surgery. A recent study of acute and chronic calculous cholecystitis also found that TNF-α was increased in acute disease and that higher TNF-α was associated with longer hospitalization [18]. Our finding of higher TNF-α in Grade III disease and its positive relationship with hospital stay is therefore biologically and clinically reasonable.

IL-10 showed a different but related pattern. Its level also increased with disease severity, but the association was weaker than that of IL-6 and TNF-α. IL-10 is mainly an anti-inflammatory cytokine and may increase as a regulatory response to strong inflammation. Previous research has reported higher IL-10 levels in moderate-to-severe acute calculous cholecystitis and identified IL-10, together with IL-6, as an independent factor related to disease severity [16]. Thus, the increase in IL-10 in our severe group may represent an attempt by the body to control the strong inflammatory response.

The operative findings were also consistent with the biomarker results. Patients with higher severity grades had longer operative time, more postoperative complications, and longer hospital stay. Conversion to open surgery was also more frequent in severe disease, although this difference did not reach statistical significance. Severe inflammation can cause edema, adhesions, fibrosis, and difficulty in identifying normal anatomy. The Tokyo Guidelines also recognize that increasing severity can make laparoscopic cholecystectomy more difficult and may require a bailout procedure or conversion in selected cases. The significant association of severity grade with hospital stay and complications in our study is also in line with previous validation of the Tokyo grading system [19].

The multivariable analysis is particularly important because age, diabetes, hypertension, smoking, and other factors may influence inflammatory markers [20]. After adjustment, ferritin, IL-6, and TNF-α remained independently associated with severe acute cholecystitis. IL-6 showed the strongest association, followed by ferritin and TNF-α. This suggests that the relationship was not explained only by age, comorbidities, or duration of symptoms. However, these findings should be considered as an association and not proof of a causal relationship.

From a clinical point of view, serum ferritin and cytokine testing may provide additional information when the clinical picture is not clear. Ferritin is already commonly measured in clinical laboratories, while cytokine testing may be less practical because of cost and laboratory requirements [21]. However, the present study did not assess whether ferritin or cytokine testing provides better diagnostic or prognostic performance than routine markers. Therefore, these biomarkers should not be considered a replacement for CRP or leukocyte count. Their value in the present study is mainly related to their association with increasing disease severity. Among the studied cytokines, IL-6 showed a strong association with disease severity. However, this finding alone is not sufficient to recommend IL-6 for routine clinical screening. Its additional value over conventional inflammatory markers needs to be confirmed in prospective studies.

Limitations

This study has some limitations. It was conducted at a single centre with a relatively small sample size. The study period was also short. Cytokines were measured at one preoperative time point, so their changes after treatment could not be assessed. Ferritin can also be affected by conditions other than acute inflammation, although major inflammatory and chronic diseases were excluded. The study did not directly compare the diagnostic or prognostic performance of ferritin and cytokines with conventional markers such as CRP and total leukocyte count. Therefore, the findings show an association with disease severity but do not establish an additional clinical benefit of routine biomarker testing. Further multicentre studies with larger samples are needed to confirm these findings. Future research can also assess the diagnostic cut-off values and combined predictive value of ferritin, IL-6, TNF-α, CRP, and other routine markers.

## Conclusions

Serum ferritin, IL-6, and TNF-α were significantly higher in patients with more severe acute cholecystitis and remained independently associated with severe disease after adjustment for important confounders. IL-6 showed the strongest relationship with severity. Ferritin also showed a clear association with inflammatory markers and clinical outcomes. These findings suggest that ferritin and cytokine profiles may have a useful role in assessing the severity of acute cholecystitis and identifying patients who may require closer monitoring and more careful surgical planning.
